# Forthcoming event

**DOI:** 10.1155/S1463924602000068

**Published:** 2002


Journal of Automated Methods & Management in Chemistry
Vol. 24, No. 1 (January–February 2002) pp. 29–30

Taylor & Francis

Taylor & Francis Group

### Forthcoming event

### Analytica 2002—Robots and biochips: *ANALYTICA Forum* focuses on practical uses

With more than 45 lectures and presentations, the *ANALYTICA Forum* will feature an unsurpassed program of information events covering everything from new products for laboratory automation and the use of IT and Internet systems in the pharmaceuticals industry to lab-on-chip solutions.

ANALYTICA will take the dynamic growth of products and solutions for laboratory technology, diagnostics and analysis into account at the *ANALYTICA Forum*. Industry experts and service providers will introduce the latest developments and discuss their benefits and potential applications with an audience of industry professionals. The trends and highlights of the *ANALYTICA Forum* at a glance: automated lab-on-chip systems, laboratory robots and Internet solutions.

Lab-on-chip—the sector for identifying pathogens in food or proving that plants have been genetically altered—is changing at a rapid pace. What could only be performed manually and required several tedious steps just a few years ago is now considered a routine task for automated biochip analysis systems. Several suppliers will focus on this recent trend at the *ANALYTICA Forum*. The forum, which will be held in Hall B3 at ANALYTICA in Munich on 23–26 April 2002, is a regular gathering for visitors in all fields who are asking such questions as: How can I find specialized information on the Internet quickly? What is the state of the biotechnology sector in Europe? What opportunities are associated with e-commerce?

Visitors can expect an attractive program of practical information events that go far beyond mere product presentations on all four days of the fair.

### *Focusing on the latest trends: biochips*

Biological microchips, or 'biochips', were the product of collaboration between molecular biologists and microsystem engineers. Biochips are miniaturized substrates made of glass, silicon or plastics to which biomolecules have been bonded in high densities in predefined microarrays. They can react with other substances. Miniaturization of the analysis platform, the ability to perform various analyses simultaneously and the high level of automation are increasing sample throughput rates and reducing cost and time requirements.

ANALYTICA exhibitor GeneScan Europe, for example, couples biomolecules in high densities on a glass substrate. Besides oligo-nucleotides, cDNAs, PCR products and larger DNA constructs, a newly-developed polymer brush technique also makes it possible to bond peptides and proteins to the glass surface. The GMO*Chip* from GeneScan combines detection and identification testing of genetically altered organisms or components in raw materials and finished products in a single inspection. Until now it was necessary to conduct these tests separately. As a result, the inspections are quicker and less expensive for the laboratory. Besides GeneScan, other companies will also present their biochip solutions for diagnostic laboratories at the *ANALYTICA Forum*.

### *Metrology lab on a chip*

How much reagent flows through a microdosing unit? Precision testing, dosing and regulation of minute flow volumes are necessary in biotechnology and medical technology as well as in environmental analysis and automotive engineering. A new type of chip with thermosensors registers even the slightest changes. The advantage of miniature measuring systems of this kind is that they experience almost no wear and react almost immediately. The Institute of Sensors, Microfluidics and Information Technology (HSG-ISIT) in Villingen-Schwenningen will present this technology on the first morning of the *ANALYTICA Forum*.

### *Using robots for high-throughput screening*

The genome has been decoded, but we still know very little about the functions of and interaction between the individual genes. 'In many cases diseases arise when there is a malfunction in these carefully orchestrated processes', explains Professor Peter Buckel from the Munich-based biotech company Xantos. There are exactly the various disease-related gene functions that researchers at Xantos can detect. They use a specially-developed, robot-based high-throughput screening method systematically to test genes from DNA bases for defined reactions in various types of tissue. Xantos will introduce its screening cascade, which is currently being used to examine the mechanisms of cell development and programmed cell death, at the *ANALYTICA Forum* on Friday, 26 April 2002.

### *Internet in the laboratory: search for substances and structures*

How does one find safety or toxicological information about chemical compounds? What search functions and databases are available? Information service provider Chemie.DE will use examples to demonstrate how electronic information can be put to better use for everyday work. The lecture program will be rounded out by round-table discussions with prominent panel members on the state of the biotechnology sector in Europe and the opportunities associated with e-commerce for the chemistry and pharmaceuticals industries.

### *Special show on genome research*

The special shows and information events at this year's fair will also include several premieres: The GSF Research Center for Health and the Environment will present a special show on life sciences called ‘Genome Research at the Helmholtz Centers’. The GSF Research Center has invited Germany’s Helmholtz Centers to present the results of their work and their genome research activities for the health and environmental sectors within the scope of a joint exhibit for the event. There are a total of 15 Helmholtz Centers in Germany whose activities focus on areas of preventative research that are important to German society. They include health, earth and the environment, energy space and transportation, the structure of matter and key technologies. The Helmholtz Society is the largest scientific organization in Germany. Within the scope of this research network, the GSF Research Center studies the complex systems of life that play a role in the debate between environmental influence and genetic predisposition. As a result, it integrates ecological and biomedical research in a common approach.

*Industry workshop for practical analysis in clinical laboratories*

The German Institute for Clinical Chemistry (DGKC) is organizing an industry workshop for the diagnostics sector that will be held at the same time as the *ANALYTICA Conference*, on 23–25 April 2002. The workshop addresses physicians, laboratory employees and scientists who work in medical laboratories and will focus on topics related to laboratory diagnostics in university clinics, hospitals and the residents’ sector. According to the DGKC, all the important manufacturers in the diagnostics sector are planning to attend the workshop.

ANALYTICA, the International Trade Fair and the *ANALYTICA Conference*, is the most important European platform for instrumental analysis, diagnostics, laboratory technology and biotechnology. It is held every two years and will open its gates at the New Munich Trade Fair Centre for the 18th time on 23–26 April 2002. Approximately 1000 exhibitors and 30 000 trade visitors are expected. Additional fair-related information and an agenda for the related-events programme are available on the Internet at www.analytica.de

*For further information, journalists please contact: Ellen Richter-Maierhofer, ANALYTICA Press Office, Messe München GmbH, 81823 München, Germany, Tel: +49(89) 949-20650, Fax: +49(89) 949-20659, e-mail: richter-maierhofer@messe-muenchen.de*

*For further information, exhibitors please contact: Dr Volker Schwartz, ANALYTICA Exhibition Manager, Messe München GmbH, 81823 München, Germany, Tel: +49(89) 949-20380, Fax: +49(89) 949-20389, e-mail: schwartz@messe-muenchen.de*

